# Treatment readiness and prognosis for problematic smartphone use: Evaluation of the Stages of Change, Readiness, and Treatment Eagerness Scale (SOCRATES) and log data

**DOI:** 10.1002/pcn5.172

**Published:** 2024-02-13

**Authors:** Nanase Kobayashi, Daisuke Jitoku, Ryoko Mochimatsu, Toshitaka Hamamura, Masaru Honjo, Shunsuke Takagi, Genichi Sugihara, Hidehiko Takahashi

**Affiliations:** ^1^ Department of Psychiatry and Behavioral Sciences, Graduate School of Medical and Dental Sciences Tokyo Medical and Dental University Tokyo Japan; ^2^ Joint Research Department of Cyberpsychiatry, Graduate School of Medical and Dental Sciences Tokyo Medical and Dental University Tokyo Japan; ^3^ KDDI Research, Inc. Fujimino‐shi Japan; ^4^ Center for Brain Integration Research Tokyo Medical and Dental University Tokyo Japan

**Keywords:** problematic smartphone use, smartphone application, smartphone log, SOCRATES, treatment readiness

## Abstract

**Aim:**

While moderate smartphone use contributes to information gathering and relationship building, excessive smartphone use, also referred to as problematic smartphone use (PSU), has raised concerns because of its addictive nature and associated health consequences. This study aimed to investigate the relationship between treatment readiness and prognosis in individuals with PSU and to assess the predictive ability of smartphone log data in evaluating treatment readiness.

**Methods:**

A sample of 47 patients with PSU participated in this study. Treatment readiness was assessed using the Stages of Change, Readiness, and Treatment Eagerness Scale (SOCRATES), and log data were collected using a smartphone log application.

**Results:**

The results showed a significant correlation between baseline SOCRATES scores and the difference in Global Assessment of Functioning scores between baseline and 6 months (Spearman's *ρ* = 0.640, P‐value = .001), suggesting that treatment readiness may explain part of the treatment outcomes (Pearson's *r* ^2^ = 0.379, P‐value = 0.032). In addition, baseline log data, including the log acquisition rate, showed a positive correlation with treatment readiness (Spearman's *ρ* = 0.328, P‐value = 0.045).

**Conclusion:**

These findings provide valuable insights into the relationship between treatment readiness and clinical outcomes in patients with PSU, and suggest the potential of log data as objective indicators of treatment motivation.

## Graphical abstract

A study of 47 problematic smartphone use patients using smartphone log data found a significant correlation between treatment readiness and improved clinical functioning after 6 months, and indicted the potential of smartphone log data as objective indicators of treatment motivation.

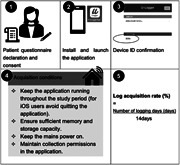

## INTRODUCTION

The widespread use of smartphones has raised concerns about excessive smartphone use.^1^ While most individuals use their smartphones adaptively and “moderate” screen time has been reported to have no negative impact on friendships,^2^ some engage in problematic smartphone use (PSU). PSU can be conceptualized as a behavioral addiction and defined as excessive use resulting in impairments,^3^ although this has been debated (Panova & Carbonell, 2018). PSU has a relatively high estimated prevalence of 12%–28% among students,^1^ which may be related to easy access to the Internet regardless of time and location.^4^ PSU can cause a variety of problems, including sleep disturbance, mental health issues such as anxiety and depression, lost academic opportunities, economic loss, accidents from walking while using smartphones, and health issues such as “smartphone neck” and headaches.^4^, ^5^

PSU shares common characteristics with substance and behavioral addictions, such as impaired impulse control, compulsive use, and neuroimaging findings (i.e., reduced volume of the orbitofrontal cortex).^6^ This implies that successful treatment of PSU, like treatment of other addictions, requires the individual to be willing to change and actively engage in their treatment.^7^ It is therefore crucial to assess the individual's motivation and readiness accurately before treatment because it is vital in predicting treatment response. Patients with higher motivation and readiness before treatment have better outcomes and higher long‐term adherence rates for substance use disorders.^8^ However, to the best of our knowledge, no study has investigated whether treatment readiness can predict outcomes in patients with PSU.

Despite the importance of accurately assessing an individual's motivation and readiness before PSU treatment, no reliable or valid tools or objective indicators are currently available. For instance, self‐reports of smartphone use tend to underestimate actual usage time.^9^ In this regard, logging apps, which are implemented on smartphones, are expected to be useful in assessing motivation and readiness because they can provide objective and accurate data without the potential for biased responses. However, to the best of our knowledge, most previous studies on log data have focused on healthy individuals, and studies on PSU using logging apps are scarce.^10^, ^11^

This study aimed to address the following clinical issues. First, to establish a positive correlation between treatment readiness and prognosis in individuals diagnosed with PSU. Second, to test the association between smartphone log data and treatment readiness among individuals with PSU.

## METHODS

### Study design

We screened 300 patients with smartphone usage problems who visited the specialized outpatient clinic of the Department of Psychiatry at Tokyo Medical and Dental University between September 2019 and June 2022. Patients were deemed eligible if they were at the age of enrolling in junior high school or older (over 12 years old). They were diagnosed by two certified psychiatrists based on the Diagnostic and Statistical Manual of Mental Disorders, Fifth Edition (DSM‐5) Internet Gaming Disorder (IGD) research criteria as a reference, with the “Internet gaming” part read as “smartphone.” The participants agreed to install a log application on their smartphones to monitor the time spent using their smartphone. Patients were excluded if they refused to participate in the study, had significantly unstable psychiatric symptoms or physical conditions that prevented their participation, or were unable to continue with outpatient visits.

**Figure 1:**
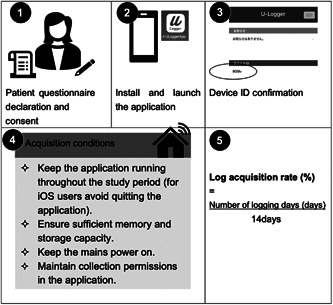
How to use the U‐Logger application.

**Figure 2:**
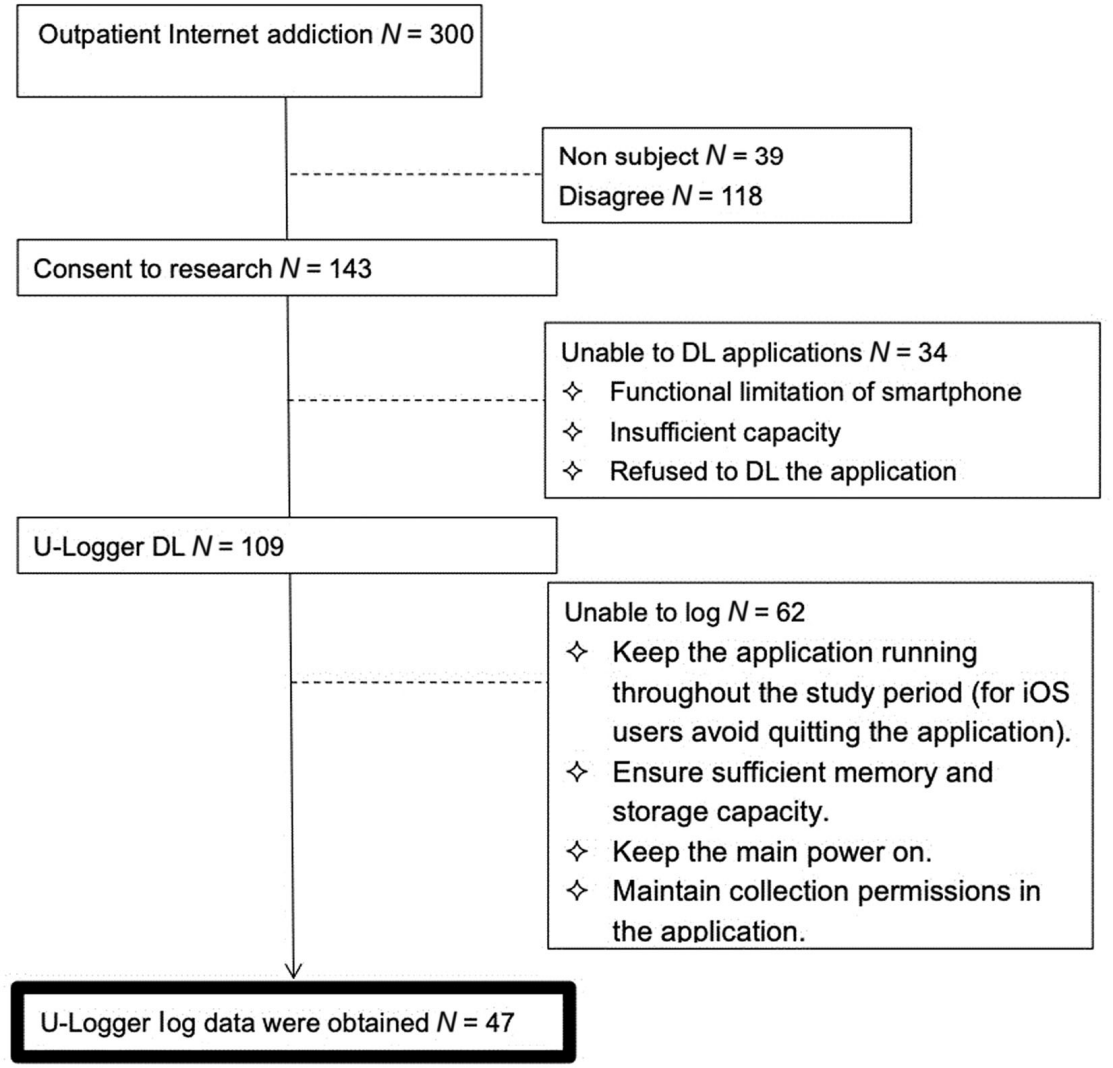
Participant selection. DL = download.

Eligible patients (*n* = 143) were asked to provide consent to participate in the observational study. After completing the baseline questionnaire administered by the interviewer, participants were asked to install the U‐Logger application for smartphone logs (Figure 1). Of the 143 registered participants, 109 completed the installation, and log data were successfully obtained from 47 participants and included in the analysis (Figure 2). All procedures followed were in accordance with the ethical standards of the responsible committee on human experimentation (institutional and national) and with the Helsinki Declaration of 1975, as revised in 2000.^5^ The study received the approval of the Ethics Committee of Tokyo Medical and Dental University (M2020‐073), and all participants and their parents provided written informed consent prior to participation.

At the initial visit, a certified psychiatrist assessed the patient's Internet use and related problems, and the individual's overall level of functioning based on social, occupational, and psychological factors using the Global Assessment of Functioning (GAF) score. Participants completed questionnaires on Internet use and treatment readiness, as described below. Log data from their smartphones were collected as baseline data for 2 weeks after download. In addition, two certified psychiatrists assessed the comorbidity of gaming disorder according to International Classification of Diseases, Eleventh Revision (ICD‐11) criteria and attention deficit/hyperactivity disorder (ADHD) and autism spectrum disorder (ASD) according to DSM‐5 criteria. At the 6‐month follow‐up, self‐administered scales, log data, and GAF scores were obtained.

### Assessments

#### Usage of smartphone, Internet, and gaming

We conducted a survey in which participants were asked about symptoms associated with smartphone, Internet, and gaming usage, including sleep disturbance, interpersonal violence, property violence, decreased motivation, lack of hygiene, and eating problems. We also collected data on the participants' Internet use patterns and habits during weekdays and weekends outside of work. To assess participants' smartphone, Internet, and gaming usage, we employed self‐administered scales: the Smartphone Addiction Scale–Short Version (SAS‐SV), the Young's Internet Addiction Test (IAT), and the Ten‐Item Internet Gaming Disorder Test (IGDT‐10), respectively. The SAS‐SV was originally developed by Kwon et al.^12^ as a screening tool for adolescents and was subsequently translated into Japanese by Tateno et al.^13^ with a slight modification. The SAS‐SV scores ranged from 10 to 60. A higher SAS‐SV score indicated problems with smartphone use. The IAT consists of 20 items related to Internet overuse, with total scores ranging from 20 to 100 points. A higher IAT score indicates more problems with Internet use. The reliability and validity of the Japanese version of the IAT have been established.^14^ The IGDT‐10^15^ is a self‐report screening scale, comprising 10 questions that assess Internet Gaming Disorder using a three‐point Likert scale (1 = never, 2 = sometimes, 3 = often). Only responses marked as “often” were considered as meeting the criterion and awarded 1 point. A higher IGDT‐10 score indicated the presence of an Internet gaming disorder. In the study by Mihara et al.^16^ about validation of IGDT‐10 based on the clinical diagnosis of IGD in Japan, a less stringent scoring method where responses of either “often” or “sometimes” were regarded as affirmative (modified version) for the Japanese version of IGDT‐10 demonstrated higher sensitivity compared to the original scoring method. The Mihara version was employed in this study because the subjects were Japanese.

#### Treatment readiness to change

The Stages of Change, Readiness, and Treatment Eagerness Scale (SOCRATES)^10^ was used to assess the participants' readiness to change their Internet and smartphone usage at baseline. The scale, developed by Miller and Tonigan,^10^ is commonly used to assess the outcome of non‐pharmacological approaches to the treating addictive disorders, assessing the degree of problem recognition and the necessity of receiving support. The Japanese version of the SOCRATES was developed and validated by Kobayashi et al.^17^ The scale was originally developed for alcoholism, but has been used for a wide range of addictive disorders, including drug addiction^17^ and gaming disorders.^18^

The SOCRATES consists of 19 items. Responses are scored on a five‐point Likert scale from 1 (strongly disagree) to 5 (strongly agree) and summed for each of the following domains: recognition, ambivalence, and taking action. Recognition assesses whether the person acknowledges their addictive behavior and is eager to change. Ambivalence measures the extent to which people are aspirational, with or without the influence of the addictive behavior. Staging estimates self‐efficacy and assesses the extent to which people take action to stop their addictive behavior. This domain is known to be a predictor of cessation or reduction of addictive behaviour.^18^

#### Social function (GAF score)

The GAF score was used to evaluate social function.^19^ The GAF score is widely used to quantitatively evaluate the overall functioning of individuals with mental disorders. It uses a scale ranging from 0 to 100, with higher scores indicating higher levels of functioning. The GAF score considers various factors related to a patient's overall functioning, including medical history, psychiatric symptoms, social functioning, and the presence of problematic behaviors.^19^

#### The U‐Logger application

U‐Logger, a smartphone logging application developed by the KDDI Corporation and KDDI Research, Inc. (Saitama, Japan), was used to record the log data. The application is not commercially available and was developed specifically for this study. The application is designed to operate in the background without disrupting the normal smartphone usage. When the application was downloaded, a device ID was randomly assigned for identification. The application records smartphone behaviors, such as power status, program activation, and screen on/off notifications. Importantly, the application did not access personal information such as photos or message content. The collected data were stored on a log storage server. Participants were instructed to keep the application running throughout the study period, avoid quitting the application (for iOS users), ensure sufficient memory and storage capacity, maintain mains power, and accept collection permissions in the application.

We used data on the number of days logged during the 2‐week baseline and screen time during the night (midnight to 6 a.m.). Screen time was measured based on screen time on/off and application launches. The log acquisition rate was calculated as the percentage of days during the 2‐week period after application installation in which logs were successfully retrieved. Medications for comorbidities, cognitive‐behavioral therapy, and a day care program were provided for 6 months. A follow‐up assessment was conducted 6 months after baseline, and log data from 2 weeks before and after each assessment point were calculated in the same manner as at baseline.

### Statistics analyses

To determine whether treatment readiness was related to treatment outcomes in PSU, we examined the Spearman correlation between baseline SOCRATES and ΔGAF score (the difference between the GAF score at 6 months and the baseline GAF score). Next, to determine whether treatment readiness for PSU could be predicted from log data, we conducted a correlation analysis of baseline SOCRATES and smartphone log data, including acquisition rates. Furthermore, correlation analyses were performed for factors related to the SOCRATES and smartphone log acquisition rates. Specifically, the baseline log data, IAT, SAS‐SV, and IGDT‐10 were determined for their association with the baseline assessment of readiness to treat (SOCRATES). SPSS version 27 (IBM Corp.) was used for all analyses. P‐values less than .05 were considered to be statistically significant.

## RESULTS

### Characteristics of the sample

The patient demographics are summarized in Table 1. The majority of the participants were male (70%) and exhibited a strong inclination towards gaming. The breakdown of most frequently used content was 57% games, 19% social media, 15% video, 4% internet browsers, and the remainder other. Many of the patients who mainly used social media and video played games for long periods of time. Overall 81% of participants were diagnosed with gaming disorder in ICD‐11. Among all patients, 36% had comorbid ADHD and 28% had comorbid ASD. The primary concerns expressed by the patients and their families included sleep disturbance, violent behavior, decreased motivation, lack of hygiene, and dietary problems.

**Table 1:** Sample characteristics (*n* = 47).

Demographic data	*N* (%)/median (min–max)
Sex	
Male	33 (70%)
Female	14 (30%)
Age (years)	
12–19	32 (68%)
20–29	13 (28%)
Over 30	2 (4%)
*Diagnosis, physician evaluation*	*N (%)/median (min–max)*
Gaming disorder	36 (81%)
ADHD	17 (36%)
ASD	13 (28%)
Duration of illness (month)	24 (2–144)
GAF	48 (30–70)
*Symptoms*	*N (%)*
Sleep disturbance	37 (79%)
Interpersonal violence	8 (17%)
Violence against property	19 (40%)
Decreased motivation	35 (74%)
Lack of hygiene	19 (40%)
Dietary problems	25 (53%)
*Self‐administered scales (baseline)*	*Median (min–max)*
Screen time	
Weekday (h)	8 (0–17)
Weekend/holiday (h)	10 (2–18)
SOCRATES	
Recognition	27 (12–35)
Ambivalence	13 (7–18)
Taking steps	20 (9–33)
IAT	65 (33–96)
SAS‐SV	38 (17–54)
IGDT‐10	6.5 (0–9)
*Log data*	*Median (min–max)*
Baseline log acquisition rate (%)	36 (7–93)
Baseline average nighttime screen time (s)	5270 (2–21600)

*Note*: Sleep disturbances = difficulty falling asleep, waking up in the middle of the day, daytime sleepiness; lack of hygiene = not taking a bath, not brushing teeth, not throwing out the garbage; dietary problems = overeating at night, skipping meals due to excessive gaming, rather than disorders related to body image, such as anorexia nervosa; SOCRATES = Stage of Change Readiness and Treatment Eagerness Scale (low value: recognition ≦30, ambivalence ≦13, taking steps ≦30; IAT = Internet Addiction Scale (cutoff ≧40); SAS‐SV = Smartphone Addiction Scale Short Version (cutoff ≧33); IGDT‐10 = Internet Gaming Disorder Test 10 (cutoff ≧5); GAF score = Global Assessment of Functioning. Average nighttime screen time = average screen time during the night (midnight‐6 a.m.) divided by the number of days logged.

Abbreviations: ADHD, attention deficit/hyperactivity disorder; ASD, autism spectrum disorder.

The age distribution of the patients was as follows: 68% were in their teens, 27% in their 20s, and the remaining 5% were older. In addition, 91% were students aged 25 or younger, and there was only one patient each in the age group of 26 and older. The duration of illness ranged from 2 months to 12 years. The median screen time in this study was higher, ranging from 8 to 10 h, compared to the findings of a 2021 survey conducted in Japan, which reported that teenagers spend less than 2 h per day on smartphones, and individuals in their 20s spend approximately 3.5 h.^20^ However, patients who had been subjected to strict parental restrictions on smartphone usage prior to their outpatient visit reported a maximum Internet usage of 2 h. The median scores for the IAT, SAS‐SV, and IGDT‐10 exceeded the cut‐off values, indicating notable addiction tendencies. Readiness to change, as assessed by the SOCRATES, had low scores for recognition (27 points), ambivalence (13 points), and taking action (20.5 points). The response rates for all self‐administered psychological scales at the 6‐month follow‐up were low, at 38%, and were therefore excluded from the analysis (Table S1).

### Smartphone logs by smartphone application

The log acquisition rate represents the percentage of days logged within 2 weeks of application installation, and participants who were able to log for at least 1 day were included in the analysis. The median was 36% (range 7%–93%). The average night time screen time was calculated as the total screen time during the night (0:00 a.m.–6:00 a.m.) divided by the number of days logged, with a median of 5270 s (2–21,600 s), that is, a median of 1.46 h and a maximum of 6 h, so some subjects used their phones at night without sleeping at all.

**Figure 3:**
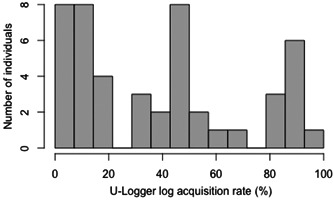
The distribution of log acquisition rate. The following table shows the distribution of the log acquisition rates for smartphones. Rates of 0% are excluded.

Figure 3 shows the distribution of smartphone logging rates; less than one‐third of the participants logged more than 50% (1 week). This means that either the app was deleted during study participation or there was insufficient capacity on the smartphone to run the application. The 6‐month follow‐up log data were excluded from the analysis because of the small amount of data obtained (42%) (Table S1).

### The correlation between readiness to change of PSU and prognosis

We examined the correlations between readiness to change at the initial visit and changes in social functioning (∆GAF score; 6‐month follow‐up GAF – baseline GAF) (Table 2). Our results showed significant positive correlations between the ∆GAF score and SOCRATES‐recognition (Spearman's *ρ* = 0.550, P‐value = .008), SOCRATES‐ambivalence (Spearman's *ρ* = 0.601, P‐value = .003), and SOCRATES‐total (Spearman's *ρ* = 0.640, P‐value = .001).

**Table 2:** The correlation between readiness to change PSU and prognosis.

	ΔGAF score
SOCRATES (recognition)	0.550*
SOCRATES (ambivalence)	0.601*
SOCRATES (taking action)	0.201
SOCRATES (total)	0.640*

*Note*: Our results showed significant positive correlations between the ∆GAF score and SOCRATES (recognition, ambivalence, total).

* P < 0.05.

Multiple regression analysis was performed for ΔGAF with SOCRATES (recognition, ambivalence, total). No collinearity problems with variance inflation factors of 10 or less for all variables. The multiple linear regression model demonstrated that SOCRATES predicted the ΔGAF score, *F* = 3.669 (Pearson's *r* ^*2*^ = 0.379, P‐value = .032). SOCRATES (recognition, ambivalence, total) taken together were responsible for 37.9% of the explained variability in ΔGAF score. Scales related to Internet and gaming were partially correlated with SOCRATES‐recognition, but not with total SOCRATES scores or ΔGAF (Table S2).

### Relationship between readiness to change and log acquisition rate

**Figure 4:**
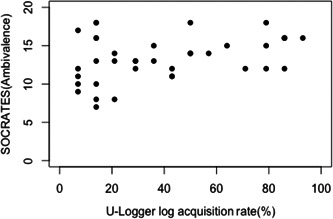
SOCRATES (Ambivalence) and log acquisition rate. Scatterplot showing the positive correlation between log acquisition rate and SOCRATES‐Ambivalence.

To examine the association between treatment readiness and log acquisition rates, we performed a correlation analysis between SOCRATES and log acquisition rates. Correlation analysis revealed that the relationship between readiness to change as measured by the SOCRATES subscale and log acquisition rate was SOCRATES‐recognition (Spearman's *ρ* = 0.317, P‐value = .053), SOCRATES‐ambivalence (Spearman's *ρ* = 0.328, P‐value = .045), SOCRATES‐taking action (Spearman's *ρ* = −0.158, P‐value = .344), and SOCRATES‐total (Spearman's *ρ* = 0.153, P‐value = 0.358), showing a positive correlation between SOCRATES‐ambivalence and log acquisition rate (Figure 4).

### Analysis of younger age groups

Due to significant variability in age distribution, we excluded age groups with a sample size of one or less to ensure statistically reliable analysis. As a result, our dataset comprised 43 individuals aged 25 or younger. The analysis of this dataset yielded the following results. In the correlation between SOCRATES scores and ΔGAF, similar to the original findings, significant correlations were observed for all but SOCRATES‐taking action. Notably, SOCRATES‐recognition (Spearman's *ρ* = 0.567, P‐value = 0.007), SOCRATES‐ambivalence (Spearman's *ρ* = 0.620, P‐value = .003), and SOCRATES‐total (Spearman's *ρ* = 0.616, P‐value = .003) showed significant correlations. Additionally, in the correlation between log acquisition rate and SOCRATES scores, the results mirrored our original findings, with significant correlations not only in SOCRATES‐ambivalence (Spearman's *ρ* = 0.388, *i*‐value = .019) but also in SOCRATES‐recognition (Spearman's *ρ* = 0.372, P‐value = .025).

## DISCUSSION

In this study, we demonstrated significant associations between treatment readiness, as assessed by SOCRATES, and the prognosis of PSU. Moreover, the acquisition rate of smartphone logs was associated with treatment readiness.

### Predictors of prognosis from readiness to change

This study provides the first confirmation of the prognostic value of the SOCRATES tool in predicting outcomes, specifically in the PSU population, while previous research has established the prognostic value of predicting outcomes in substance use disorder.^11^ The results revealed a robust positive correlation between total SOCRATES scores and the degree of change in GAF scores from baseline to 6 months, indicating that higher SOCRATES scores were consistently associated with more significant improvements in social functioning over the course of treatment. These findings suggest a potential utility of the SOCRATES tool for prognosticating outcomes in populations with PSU.

### Association between smartphone logs and readiness to change

We observed an association between the acquisition rate of smartphone logs and treatment readiness. Individuals with high SOCRATES‐ambivalence subscores demonstrate a growing concern for their problematic behaviors, indicating an awareness of the addictive behaviors and the social issues associated with them. Ambivalence, which can hamper treatment progress, is a crucial factor in the addictive behaviors and cravings observed in alcohol and gambling disorders.^21^ Resolving ambivalence is indeed one of the objectives in motivational interviewing.^22^ The rate at which individuals acquire logs through their smartphones serves as an indicator of their engagement in self‐monitoring their own behaviors and habits. This study highlights the potential usefulness of log visualization as a supportive treatment tool.

Contrary to our initial expectations, we did not find a significant correlation between the SOCRATES‐taking action subscores and prognosis. Previous research has indicated that in cases where addictive behaviors were already declining before the baseline assessment, short‐term follow‐up may lead to a worsening of these behaviors compared with baseline.^7^ Given the intricate nature of behavioral change and its susceptibility to relapse, it is crucial to avoid premature discontinuation of treatment.^11^ Consequently, to effectively provide tailored support, it is imperative to gather insights into the underlying psychological phenomena and strategies that impede progress and contribute to behavioral reversal.

### Limitation

While this study provides valuable insights into the prognostic value and utility of the SOCRATES tool and smartphone logs in a population with PSU and substance use disorders, it is important to acknowledge several limitations that should be considered when interpreting the findings. First, 37.9% of the change in ΔGAF scores after 6 months of treatment was attributable to pre‐treatment SOCRATES, suggesting that pre‐treatment readiness plays an important role in explaining changes in social functioning over the course of treatment, but it should also be noted that there are other factors that were not examined in this study. Various factors could have influenced the GAF scores at follow‐up, and a more comprehensive assessment for PSU would ideally involve a combination of other self‐rating and/or objective scales specifically designed for behavioral addictions. In the present results, dependence scales such as IAT, SAS‐SV, and IGDT‐10 had low recovery rates and could only be assessed with GAF. The reasons for the low recovery rate of the self‐administered psychological scales after 6 months are as follows. Questionnaires were provided during outpatient visits after 6 months, and if responses were not obtained for more than a month due to loss or procrastination, we urged an immediate response at that point. If no response was obtained then, it was treated as a refusal or non‐response. There were no patients who refused to respond at the time the questionnaires were distributed. Further research is needed to fully understand these factors.

The second limitation is the quantity and quality of the sample. In our study, the log collection rate from patients was significantly lower than that of healthy college students and adults.^9^ One reason for this is that, unlike previous studies, no honorarium was paid. This was due to concerns that offering gratuities to the patient group might create new problems such as gaming charges. This limited the collection of log information, such as time and frequency of use at baseline, and thus did not provide sufficient data for analysis. Moreover, out of the 300 individuals who visited the specialized outpatient clinic, 261 were deemed to have a problem and 143 people (54% of the 261) consented to participate in the study. Of these, 109 individuals (41%) completed the installation of the app, and log data were actually obtained from 47 participants (18%). Only 20 participants (7%) were able to complete log collection over a period of 6 months. This suggests the difficulty in treating PSU.

Consequently, caution should be exercised in generalizing the findings of this study, as these individuals may not fully represent the overall characteristics of PSU. Finally, the age range of the patients in this study varied from teens to those in their 40s, resulting in a heterogeneous sample. The age distribution was skewed, with 91% of the patients being students under 25 years of age. While reanalysis of the under‐25 age group yielded almost identical main outcomes, caution is advised when applying these results to individuals aged 26 and older.

## CONCLUSION

To the best of our knowledge, this is the first study to examine the utility of SOCRATES and smartphone logs to assess treatment readiness in PSU patients. Incorporating these assessment measures could help healthcare professionals predict treatment outcomes and provide appropriate support and interventions. Although further research is warranted, these findings hold promise for informing clinical approaches and refining treatment plans in the context of problematic smartphone use.

## AUTHOR CONTRIBUTIONS

Conceptualization: Nanase Kobayashi, Daisuke Jitoku, Toshitaka Hamamura, Masaru Honjo, Hidehiko Takahashi. Data curation: Nanase Kobayashi, Daisuke Jitoku, Ryoko Mochimatsu, Toshitaka Hamamura. Formal analysis: Nanase Kobayashi, Daisuke Jitoku, Toshitaka Hamamura, Genichi Sugihara. Funding acquisition: Nanase Kobayashi, Daisuke Jitoku, Hidehiko Takahashi. Investigation: Nanase Kobayashi, Daisuke Jitoku, Ryoko Mochimatsu, Toshitaka Hamamura. Methodology: Nanase Kobayashi, Daisuke Jitoku, Toshitaka Hamamura, Masaru Honjo, Hidehiko Takahashi. Project administration: Nanase Kobayashi, Daisuke Jitoku, Toshitaka Hamamura, Masaru Honjo, Hidehiko Takahashi. Resources: Nanase Kobayashi, Daisuke Jitoku, Ryoko Mochimatsu, Toshitaka Hamamura, Masaru Honjo. Software: Nanase Kobayashi, Daisuke Jitoku, Toshitaka Hamamura, Masaru Honjo. Supervision: Daisuke Jitoku, Hidehiko Takahashi, Toshitaka Hamamura, Masaru Honjo. Validation: Nanase Kobayashi, Daisuke Jitoku, Genichi Sugihara, Shunsuke Takagi. Visualization: Nanase Kobayashi, Daisuke Jitoku. Writing—original draft: Nanase Kobayashi, Daisuke Jitoku, Genichi Sugihara. Writing—review and editing: Daisuke Jitoku, Toshitaka Hamamura, Masaru Honjo, Shunsuke Takagi, Genichi Sugihara, Hidehiko Takahashi.

## CONFLICT OF INTEREST STATEMENT

Nanase Kobayashi, Daisuke Jitoku, Ryoko Mochimatsu, Shunsuke Takagi, Genichi Sugihara, and Hidehiko Takahashi report equipment (smartphone log application) provided by the KDDI Corporation and KDDI Research, Inc. Toshitaka Hamamura and Masaru Honjo report financial support provided by KDDI Research. Toshitaka Hamamura and Masaru Honjo report a relationship with KDDI Research, Inc. that includes employment.

## ETHICS APPROVAL STATEMENT

All procedures followed were in accordance with the ethical standards of the responsible committee on human experimentation (institutional and national) and with the Helsinki Declaration of 1975, as revised in 2000.^5^ The study received the approval of the Ethics Committee of Tokyo Medical and Dental University (M2020‐073), and all participants and their parents provided written informed consent prior to participation.

## PATIENT CONSENT STATEMENT

Informed consent was obtained from all individuals who participated in the study. Minors also obtained informed consent from their legal guardians.

## CLINICAL TRIAL REGISTRATION

N/A.

## Supporting information

Supporting information.

Supporting information.

## Data Availability

Raw data were collected at Tokyo Medical and Dental University. Derived data supporting the findings of this study are available from the corresponding author on request.
