# Supplementary material for: Treatment readiness and prognosis for problematic smartphone use: Evaluation of the Stages of Change, Readiness, and Treatment Eagerness Scale (SOCRATES) and log data
Source: PCN Rep. 2024 Feb 13;3(1):e172. doi: 10.1002/pcn5.172 (PMC11114430; doi:10.1002/pcn5.172)
Supplement: Supplementary file 1 — Supporting information. [file PCN5-3-e172-s002.docx]

**Supplementary Table 1. Collection rate of self-administered psychological scales**

| **Baseline** | | IAT | SASSV | IGDT10 | SOCRATES  (Recognition) | SOCRATES (Ambivalence) | SOCRATES (Taking action) | SOCRATES(Total) | log |
| --- | --- | --- | --- | --- | --- | --- | --- | --- | --- |
| Frequency | valid | 40 | 38 | 39 | 38 | 38 | 38 | 38 | 47 |
|  | Missing | 7 | 9 | 8 | 9 | 9 | 9 | 9 | 0 |
|  | % | 85 | 81 | 83 | 81 | 81 | 81 | 81 | 100 |
| Median | | 65 | 38 | 6.5 | 27 | 13 | 20.5 | 62 | 29 |
| Minimum | | 33 | 17 | 0 | 12 | 7 | 9 | 34 | 4 |
| Maximum | | 96 | 54 | 9 | 35 | 18 | 33 | 78 | 93 |
|  |  |  |  |  |  |  |  |  |  |
|  |  |  |  |  |  |  |  |  |  |
| **6M follow up** | | IAT | SASSV | IGDT10 | SOCRATES (Recognition) | SOCRATES (Ambivalence) | SOCRATES (Taking action) | SOCRATES (Total) | log |
| Frequency | valid | 18 | 18 | 18 | 18 | 18 | 18 | 18 | 20 |
|  | Missing | 29 | 29 | 29 | 29 | 29 | 29 | 29 | 27 |
|  | % | 38 | 38 | 38 | 38 | 38 | 38 | 38 | 42 |
| Median | | 52.5 | 30.5 | 4 | 25 | 14 | 24.5 | 62.5 | 0 |
| Minimum | | 23 | 10 | 0 | 7 | 4 | 8 | 19 | 0 |
| Maximum | | 73 | 47 | 9 | 35 | 20 | 37 | 77 | 100 |

This table presents the collection rates of self-reported psychological scales. At baseline, the collection rate was around 80%, while at the 6-month follow-up, due to refusal or loss of questionnaires, the rate dropped to 38%.
