# Supplementary material for: Treatment readiness and prognosis for problematic smartphone use: Evaluation of the Stages of Change, Readiness, and Treatment Eagerness Scale (SOCRATES) and log data
Source: PCN Rep. 2024 Feb 13;3(1):e172. doi: 10.1002/pcn5.172 (PMC11114430; doi:10.1002/pcn5.172)
Supplement: Supplementary file 2 — Supporting information. [file PCN5-3-e172-s001.docx]

|  | IAT | SAS-SV | IGDT-10 | SOCRATES (Recognition) | SOCRATES (Ambivalence) | SOCRATES (Taking action) | SOCRATES (Total) | log acquisition rate (%) | ΔGAF |
| --- | --- | --- | --- | --- | --- | --- | --- | --- | --- |
| IAT |  | .666^**^ | .534** | .366^*^ | .310 | -.341^*^ | .069 | .360^*^ | -.040 |
| SAS-SV |  |  | .264 | .487^**^ | .247 | -.301 | .085 | .348^*^ | .091 |
| IGDT-10 |  |  |  | .043 | .145 | -.288 | -.126 | -.124 | -.096 |

**Supplementary Table 2. Correlations between measures of internet and gaming and treatment readiness, logging acquisition rate, and social functioning**

p<0.05*, p<0.01**

This table shows the presence or absence of correlations between the Internet and gaming scales, along with ratings of treatment readiness, log acquisition rate, and social dysfunction. Correlations were found between scores on the Sociological Awareness (SOCRATES-Recognition) and Addiction Scale and log acquisition rate, respectively. However, no clear correlation was found between total SOCRATES scores, changes in social functioning, and the addiction scale.
